# Optical measurements of paintings and the creation of an artwork database for authenticity

**DOI:** 10.1371/journal.pone.0171354

**Published:** 2017-02-02

**Authors:** Seonhee Hwang, Hyerin Song, Soon-Woo Cho, Chang Eun Kim, Chang-Seok Kim, Kyujung Kim

**Affiliations:** 1 Department of Advanced Circuit Interconnection, Pusan National University, Busan, Republic of Korea; 2 Department of Cogno-Mechatronics Engineering, Pusan National University, Busan, Republic of Korea; 3 Department of Fine Arts, Pusan National University, Busan, Republic of Korea; Institute of Materials Research and Engineering, SINGAPORE

## Abstract

Paintings have high cultural and commercial value, so that needs to be preserved. Many techniques have been attempted to analyze properties of paintings, including X-ray analysis and optical coherence tomography (OCT) methods, and enable conservation of paintings from forgeries. In this paper, we suggest a simple and accurate optical analysis system to protect them from counterfeit which is comprised of fiber optics reflectance spectroscopy (FORS) and line laser-based topographic analysis. The system is designed to fully cover the whole area of paintings regardless of its size for the accurate analysis. For additional assessments, a line laser-based high resolved OCT was utilized. Some forgeries were created by the experts from the three different styles of genuine paintings for the experiments. After measuring surface properties of paintings, we could observe the results from the genuine works and the forgeries have the distinctive characteristics. The forgeries could be distinguished maximally 76.5% with obtained RGB spectra by FORS and 100% by topographic analysis. Through the several executions, the reliability of the system was confirmed. We could verify that the measurement system is worthwhile for the conservation of the valuable paintings. To store the surface information of the paintings in micron scale, we created a numerical database. Consequently, we secured the databases of three different famous Korean paintings for accurate authenticity.

## Introduction

Works of art are important subjects of commercial investment owing to the increasing value of art over the years [1–4]. Works created by famous artists such as Pablo Picasso, Vincent van Gogh, Paul Gauguin, and Paul Cezanne have been sold at auction at high prices. For example, Pablo Picasso’s painting *Les Femmes d'Alger* (“Women of Algiers”) was purchased for $179.3 million in 2015. A famous work of art titled *When Will You Marry*?, created by Paul Gauguin in 1892, was recently sold at auction for $300 million, the highest price ever paid for a painting as of February 2015 [5].

The art market has enjoyed a commercial boom in recent years, and the number of art collectors has sharply increased. As interest and demand for the works of famous artists have increased, many people have unknowingly or sometimes willingly purchased forgeries. Thus, the number of forgeries has increased, and the techniques used in the creation of forgeries have also rapidly improved. The skilled forgers have made numerous forgeries that are nearly identical to the well-known works from which they were copied. This cultural trend has made it much more important to determine whether a work of art is genuine. In particular, the authenticity of famous works has been called into question all over the world [6–10]. Determining the authenticity of works could be necessary to preserve genuine works and prevent them from being switched with forgeries.

There are many techniques to authenticate works of art [7,10–24]. However, most conventional techniques have invasive and destructive data collection methods. In addition, these methods are often costly, time-consuming, complex and somewhat inaccurate [7,10,13,22,23]. A scanning electron microscope (SEM), for example, can be used to analyze features such as painted layers and pigments. However, paintings are necessarily damaged during sampling, and data can be obtained from only a very small area because of the chamber size and the need to minimize damage [12,24]. The carbon isotope dating method can be used to estimate the production year, but this method has a wide error range—approximately 50 years. This analysis method can be applied to paintings created since the late 1950s because the testing of nuclear weapons raised ^14^C concentrations [12]. Connoisseurs, who take subjective views of works, play a significant role in evaluating the authenticity of works of art, but such evaluations could induce problems due to a lack of ethics and expertise [7, 25].

The limitations are widely known, and alternative techniques may be required to compensate for these limitations [7]. Each of these typical analysis methods can measure only one characteristic such as outline, depth information or dating of painting pigments. Thus, many artists have requested a novel system for convenient, fast and low-cost differential diagnosis. Using optical coherence tomography (OCT) in painting may be one of the noticeable cases satisfy the requirements, because OCT has been well-known as a non-invasive, non-destructive and high resolved imaging method [14, 26–30]. However, it also gives only the depth information, and need to spend long time to scan the whole area of paintings.

In this study, we designed a simpler and more compact optical measurement system with high reliability for determining the authenticity of paintings. The system scans a whole area of paintings to measure the optical characteristics simultaneously. We used fiber optics reflectance spectroscopy (FORS) to measure the color characteristics of works of art. FORS has been used to classify the colors of pigments in paintings by creating a spectral database of changing wavelengths without causing damage [15, 31–34]. Furthermore, we designed the system to measure the specific topographic features of paintings using a line laser. The pigments and individual brushstrokes used in paintings are extremely diverse and can be studied as the characteristic factors of a painting. Thus, we could determine the topographic information of paintings by measuring the reflectance light from painting surfaces. To test the system, we created forgeries of famous Korean artworks that have different styles. We then used FORS and topographic data to create a numerical database that recorded the different characteristics.

## Studied artworks

**Fig 1:**
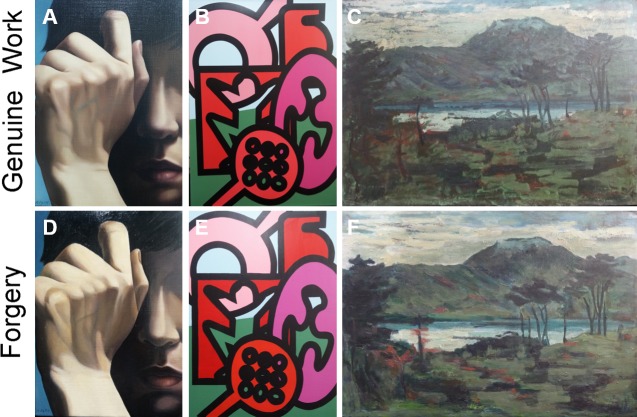
The image of paintings. The genuine works of art created by the famous Korean artists (A) *Self-portrait*, (B) *Morpheme*, and (C) *Unknown*. (D), (E), and (F) were forged for the experiments.

To test applicability, we used three genuine paintings created by famous Korean artists to analyze their optical characteristics in Fig 1A–1C. We also created three art forgeries in Fig 1D–1F to compare the optical characteristics of the genuine paintings. The features of the paintings used for experiments were the mixtures of the oil and acrylic paints, which can produce many different colors, the various consistencies of the adjusted paints, and the varying paint thicknesses. Thus, the surface roughness of the paintings and the different brushstroke styles of the artists could be determined. There are explanations of genuine paintings.

**Painting 1:** *Self-portrait* in Fig 1A was created on linen canvas (33.4 × 22.4 cm) and colored with oil paints using a graded color technique. This work of Realism was created by Jean-Ey Lee (1969–) in 2014. The inherent meaning of the painting, i.e., the existence of the ambivalent inner world of the artist, is expressed by the subject’s covering half of her face with her hands. The artist sketched the picture and painted it with a mixed glossy and matte acrylic varnish. The signature, written in Korean characters, is in the lower left corner.

**Painting 2:** *Morpheme* in Fig 1B was created on cotton canvas (53 × 40.9 cm) and colored with acrylic paint. The painting, a piece of Conceptual Art following the Neo-pop trend, was created in 2014 by Chang Eun Kim (1974–), a professor at Pusan National University. The title *Morpheme* means “the smallest unit” and implies that imagery serves in the same capacity as morphology in linguistics. The artist created a variety of formative works that combined and arranged 6 or 7 morphemes. The surface of the painting is mostly flat even though it was painted with acrylic paints because it was sandpapered repeatedly to smooth it. After painting, two coats of transparent acrylic varnish were added to prevent discoloration and damage.

**Painting 3:** *Unknown* in Fig 1C was created on cardboard (33.2 × 45.5 cm) and was colored with oil paint. It is an Impressionistic work of art created by Hye Soo Song (1913–2005) in the 1960s. The artist was one of the first-generation artists of Western-style painting and created many artworks that expressed Korean sentiments. The painting shows remarkable Impressionistic techniques such as strong and rapid brushstrokes and bold painting and expresses a lyrical feeling in its imagery of a distant mountain, river, woods, and a traditional house.

Forgeries, as shown in Fig 1D–1F, were carefully prepared for comparison with the genuine works under agreements from artists of genuine paintings. To perfectly copy the genuine works, the similar materials were assembled including the canvasses, paints and varnish [25]. Even though the painter of a forgery does his best to copy the original work, it is impossible to create two identical paintings. The paintings may have different characteristics such as topography, color, brightness and brush strokes. According to the blind test results, 70% testers could not distinguish the genuine paintings from forgeries with their naked eyes. Thus, we numerically measured the details at the micron scale, and investigated the different optical characteristics of each painting using this system.

## Methods

### Optical system

**Fig 2:**
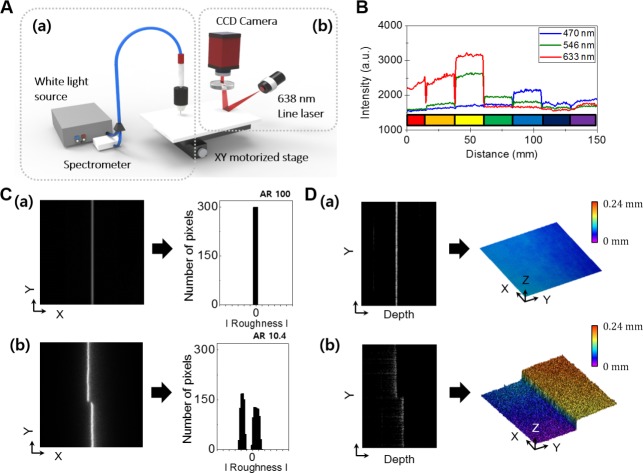
The schematic of optical measurement system and pilot experiments. (A) Schematic illustration of the system; (a) RGB measurements using a white light source and spectrometer. (b) Topographic measurements using a line laser and CCD. (B) The measured RGB spectra of the rainbow-colored image. (C) The topographic image obtained from; (a) Silver mirror. (b) Two sheets of A4 paper. (D) Tomographic image and 3-D topographic image obtained by OCT; (a) Silver mirror. (b) Two sheets of A4 paper.

Fig 2A is a schematic illustration of the experimental setup for measuring the color intensities and topographies of paintings. To obtain the color information, a UV-VIS-NIR range light source (range: 230–2500 nm, DH-2000-BAL, Ocean Optic Inc., Florida, Dunedin) was used in Fig 2A (a). The color information was obtained using a UV-VIS-NIR spectrometer (detector range: 200–1100 nm, USB-4000, Ocean Optic Inc., Florida, Dunedin). The incident light reached the surfaces of the paintings via a fiber. The light is then reflected from the surface and redirected back to a fiber that leads to the spectrometer. For accurate measurements, light should be detected over a small area. Thus, an objective lens (UPLFLN 20×, Olympus, Japan) was added to the end of the fiber to focus the light down to a diameter of 1 mm. The location of the sample was precisely controlled by a micro-motorized stage, and the paintings were scanned at a constant speed of 0.9 mm/s on the XY-motorized stage. As the scan speed decreased, the data resolution can be enhanced. It took approximately 6 hours to precisely obtain the data for the entire *Morpheme* painting in Fig 2B at a speed of 0.9 mm/s. However, the entire scanning was performed in approximately one hour at a speed of 0.9 cm/s and still showed unique specifications (S1 Fig). To prevent against dislocation problems during the measurements of the paintings, we employed a fastening device in the sample stage. In Fig 2A, part (b) shows the topography measurement system. The topography was analyzed using a line laser (Z10M18B, Z-laser, Germany) which operated with a power of ~ 10 mW at a wavelength of 638 nm, which made it possible to analyze the paintings without damaging them [35]. The 638 nm line laser was directed onto the surface at 80°. The reflected light from the surface was detected using a CCD (1600 × 1200 pixels, Pike F505B, Allied Vision Tech., Germany). The laser source was focused on the paintings using a Z-axis stage and an angle stage. This system was designed to record optical measured data in whole area of painting. The air-conditioner was utilized to make constant temperature and humidity of 18 ~ 20°C and 50% in laboratory where the optical system was set. We blew dust on the paintings before experiment to remove dust.

OCT system using line-field beam in S2 Fig performed the 3-D topological images with high-resolution to verify the distinction feasibility of roughness to verify the distinction feasibility of roughness in advance, which is based on Michelson-interferometer [36]. Line-field beam was formed by cylindrical lens and focused on the reference arm and sample arm through the convex lenses. By scanning the 14 mm of line-field beam with 1-D transverse direction and detecting the line CCD (1024 pixels, LVB101CL, Crevis, Korea), 3-D topologic image could be obtained because A-san was performed by 800 nm centered swept source [37, 38]. The transverse resolution and axial resolution were approximately 28 μm × 15 μm and 3 μm. The speed of 1-D transverse scanning was 0.6 mm/s.

### Data analysis

Because the artworks were painted in a variety of colors, we were able to obtain information on many colors over a wide wavelength range. To obtain the specific color characteristics of the paintings, we chose three wavelengths and obtained the intensities at those wavelengths. We measured the intensities at wavelengths of 470 nm, 546 nm, and 633 nm, which represent blue, green, and red. Depending on the colors of the painting, the intensities and ratios will change differently.

Paintings have specific topographical features on their surfaces. Depending on the surface roughness of a painting, light will be scattered and reflected in various directions. Thus, we measured a reflected line on the surface. The field of view for the raw images is 6.5(W) × 5(H) mm. The measured raw image data were represented as 0–255 grey scale images, and had some noise which needs to be considered. The raw image data were filtered using Gaussian filter to remove some noise, and the clear images could be obtained by a high pass filter. Thus images were transformed to binary valued images (300 × 300 pixels) for use in MATLAB. The reflected lights out of threshold were ignored to obtain region of interest (ROI) image in the detection process. The remaining data are results of topographic features represented the ROI image. We summed the pixels in every column, which were plotted in a graph, and each graph contained the roughness information. If the surface is almost flat, the graph will show low deviation. In addition, the numerical values were defined by the aspect ratio (AR) of the graph to obtain the topographic numerical data. The number of pixels at maximum point was divided by the length of pixel-exist range. If the reflected line is rough, the pixels get dispersed in wide range, and results in large number of denominator. Therefore, the AR value also decreases.

## Results

### Pilot experiments

We designed pilot experiments to determine whether it is possible to obtain information about the colors and topography of the paintings using this system as shown in Fig 2A. First, we chose a vivid rainbow-colored image to obtain spectral information about the colors. Fig 2B shows the spectral data for the rainbow-colored image. As the colors of the scanned area changed, different intensities at each wavelength were observed. As we expected, the wavelengths of 470, 546, and 633 nm stood out in the bluish, greenish, and reddish regions, respectively. The different colors of the scanned regions showed unique combinations of wavelengths. By comparing the combined properties of the colors, we obtain color information to determine authenticity. Next, for the topographic tests, images of reflected light from a silver mirror surface were obtained as a control. As indicated in Fig 2C (a), the reflected line is extremely sharp and straight. Since the silver mirror has an exceptionally low roughness, and its reflectance in the visible range is greater than 98%. The graph shown in Fig 2C (a) is obtained from reflected silver mirror image and shows very low deviation. Here, we defined aspect ratio (AR) to quantify the topographic data, and AR of the silver mirror is set to 100. As a control, we made reference samples for the roughness measurements. The actual paintings have distinctive properties of surface roughness. Therefore, the reflected light contains topographic information in each specified region and could be used to determine authenticity. For example, two sheets of 100 μm-thick A4 paper were stacked. The top sheet was shifted little to make a topographical change. The laser was focused cross the stacked line of paper. We then collected the reflected image, and the results are shown in Fig 2C (b). Because the surface of the paper was less flat than the surface of the mirror, a slight fading around the predominant reflected line was observed. The number of pixels along the |roughness| was found to be uneven. Therefore, the width of the reflected line increased where the material was raised. In addition, the two acquired lines were independent of each other because of the existence of a fault. For this reason, the roughness graph has a bifurcated shape in Fig 2C (b). In order to confirm the data, we additionally tested the same samples using an optical coherence tomography system. The obtained 2-D tomographic image and 3-D topographic image are shown in Fig 2D. As expected, we could observe the surface roughness of the silver mirror and the paper, and also clearly measured the step height due to the existence of the top sheet. The step height was measured as 100 μm, which is exactly equal to the thickness of a single A4 paper. Consequently, we acquired entire roughness data from the sample.

### RGB spectral measurements of the artworks

**Fig 3:**
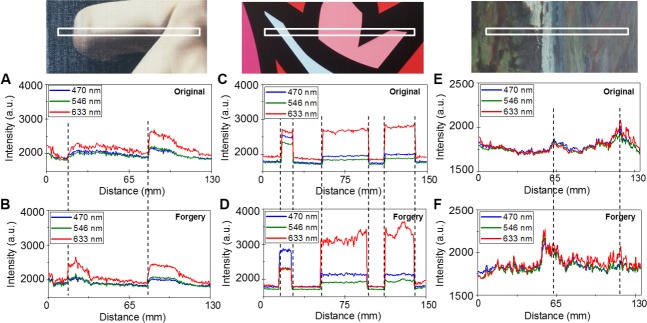
The RGB data from the genuine works and the forgeries. The RGB spectral data from the white boxes in (A) the genuine *Self-portrait* and (B) the forged *Self-portrait*, (C) the genuine *Morpheme* and (D) the forged *Morpheme*, and (E) the genuine *Unknown* and (F) the forged *Unknown*.

We measured the RGB spectra of the real artworks and obtained the color characteristics of the three pairs of genuine paintings and forgeries. We compared the scan data from multiple measurements of the same area of a painting and analyzed the conformity. In Fig 3, the white rectangular boxes indicate the scanning regions for the experiments. Fig 3A and 3B show the scanned data from *Self-portrait*. The spectra in Fig 3C and 3D are the data measured on *Morpheme*, and Fig 3E and 3F show data from *Unknown*. Fig 3A, 3C and 3E show the scan data of the genuine works, and Fig 3B, 3D and 3F show the data from the forgeries. As shown in Fig 1, the paintings appear to be similar. However, the scanning results showed obvious differences. Given that the motorized stage was used with the same operating parameters, the lengths between the featured colors are quite different when compared to the dashed lines. The intensity at 633 nm at a distance of 16 mm from the forgery was approximately 2400 a.u., which was higher than that of the original, which had an intensity of 1840 a.u.. The second prominent spectral range at distances greater than 83 mm from the forgery and the genuine work appears similar; however, the intensity of the featured region of the forgery was lower than that of the original. In Fig 3C and 3D, while the results from the genuine work showed almost equivalent intensities in the dashed lines at each color, the results of the forgery showed less uniformity. In the 633 nm wavelength spectra at distances from 55 mm to 96 mm, the maximum intensity difference in Fig 3D was 500 a.u. but, difference in Fig 3C was 200 a.u.. The spectra in Fig 3D are less smooth. The RGB spectra of the sky-blue region of the genuine work show that the red light was highly reflected (Fig 3C). When calculating the ratios of each wavelength in the genuine data in Fig 3C, the values were 34% at 470 nm, 26% at 546 nm, and 40% at 633 nm, and the 633 nm wavelength had the highest value. The forgery results, however, were 48% at 470 nm, 25% at 546 nm, and 27% at 633 nm. While the 633 nm wavelength was generally dominant in all the spectra, the 470 nm wavelength was prominent in the sky-blue region in Fig 3D. The intensities of the spectra in Fig 3E and 3F were lower than those of the other paintings because the overall colors in the white boxed region had lower chromas than the other paintings. However, the spectral intensities of the two shown in Fig 3E and 3F were quite different. The first dominant peak of the original was located at a 63 mm that is further than that of the forgery (55 mm). By comparing the RGB spectrum of the genuine work with that of the forgery, we observed differences that could not be distinguished by the naked eye.

### Topography measurements of the artworks

**Fig 4:**
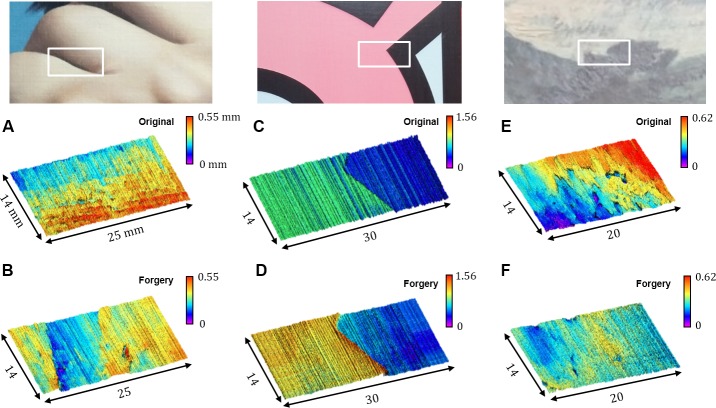
3-D topographic data using OCT. 3-D topographic images from the white boxes obtained from the genuine works and forgeries using optical coherence tomography. (A), (C), (E) are the genuine works data and (B), (D), (F) are the forgeries data.

Next, the topographic information was measured using two different measurement systems. At first, to verify that topographic information between the genuine works and forgeries are different, the precise 3-D topographic images were obtained by a high-resolved OCT system which uses the 14 mm of line-field beam as shown in Fig 4. Approximately, 14 mm × 30 mm of field of view was achieved by scanning the line-field beam laterally. Fig 4A and 4B are OCT images scanned in the white box of the genuine work and forgery of *Self-portrait*, respectively. The topological information of *Self-portrait* was less distinctive compared to the usual oil painting, however, the embossed patterns could be observed in Fig 4A. The height differences at the surface of both paintings were distributed within 0.55 mm. On the other hand, even though Fig 4C and 4D are flatter than other works, it could be more distinctive due to the layered structure which induced the height difference between the top and bottom of acrylic layer. The thickness of top layer (pink region) of the genuine work is thinner than that of forgery. Fig 4E and 4F are obtained on the genuine work and forgery of *Unknown*, respectively, which show obviously different topological results. The 3-D topographic images are significantly different because the oil paintings were greatly influenced on precise artist brushstrokes. The height difference of genuine *Unknown* was distributed within 0.62 mm while that of forgery was distributed within 0.40 mm. The OCT system provides topographic information with high resolution and high accuracy.

**Fig 5:**
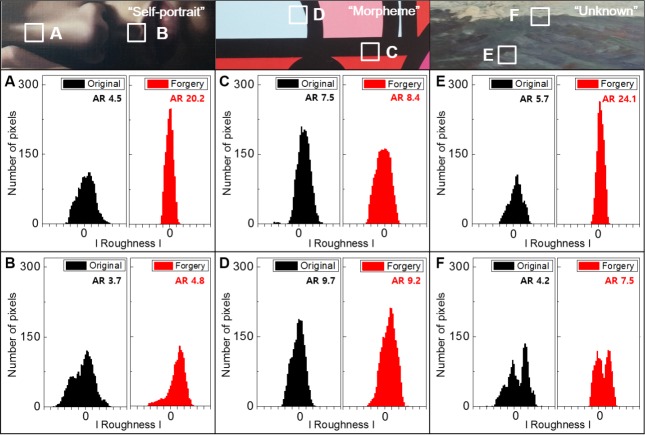
The AR data to distinguish the genuine works from the forgeries. The AR data modified from the raw topographic images obtained from the genuine works (A) *Self-portrait*, (C) *Morpheme*, and (E) *Unknown*. The data measured from the created forgeries of (B) *Self-portrait*, (D) *Morpheme*, and (F) *Unknown*. The ARs indicated on the graphs can be used to distinguish the genuine works from the forgeries (black: original work, red: forgery).

Next, we measured the topography of the same paintings with a line laser based topographic system as shown in Fig 2A. Two regions in each painting were picked randomly and scanned. For numerical comparison, the reflected line images presented in S3 Fig were modified as shown in Fig 5. The details are described in the methods. The AR of each graph, which is used to judge the deviation, is indicated in Fig 5. As we expected, rougher areas showed lower AR degrees. Fig 5A shows data from region A in the white box. The roughness of the original extended over 15 pixels and was almost twice that of the forgery. The AR degree was approximately five times larger than that of the forgery. In Fig 5B, the maximum number of pixels at the point of the maximum roughness and the roughness of the two graphs were similar; therefore, the differences in the ARs were about 1. Because the surface of *Morpheme* was flatter than those of the others, the reflected lines were rather straight. The graphs shown in Fig 5C and 5D look more similar than other results of painting, however these are definitely different. The differences of AR values in Fig 5C and 5D were 0.9 and 0.5 respectively. In case of Fig 5E, we were able to verify its authenticity easily because of the large gap in the ARs value about 20 and the shapes of the graphs. The bifurcated graphs in Fig 5F are the features in region F; however, we were able to differentiate between the original and forgery using the ARs. The graphs in Fig 5B, 5D and 5F, which we picked the worst case, may be difficult to distinguish at first. Nevertheless, the forgery can be determined using the system because there are certain differences between the genuine works and the forgeries. We confirmed that it is possible to directly distinguish the genuine works from the forgeries because the ARs and the formation of the graphs were very distinctive. The ARs of the graphs can surely provide precise information about the differences.

### Calculating the error rates for the genuine works and forgeries

**Fig 6:**
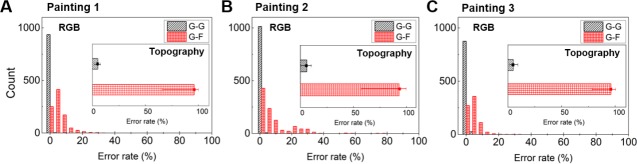
The error rates of measured data. **T**he error rates of the RGB spectra and topographic data (inset); G-G presents the error rates between the genuine works and the genuine works, G-F shows that between the genuine works and the forgeries. The error rates measured on (A) *Self-portrait*, (B) *Morpheme*, and (C) *Unknown*.

To check the accuracy of the system, we calculated the error rates in Fig 6 from the measured data. First, the intensity differences of the RGB spectra were calculated for each painting. Every time we scanned the same painting, the results were almost identical (S4 Fig). The first measured result was used as a reference for several scan results. The error rates in the genuine works are indicated as G-G, which is the black oblique line. Each bar-graph covers 4% of error range. The error rates of the G-G were within 4%, mostly under 1%, and the average error rates were 0.399% in painting 1, 0.494% in painting 2, and 0.674% in painting 3. Therefore, we were able to obtain similar results whenever scanning the paintings. On the other hand, the error rates when comparing the genuine works with the forgeries were designated by G-F. The columns are generally spread out. In Fig 6B, the error rate is up to 80%. Second, a difference in topography was calculated based on the S5 Fig data. If the location of the reflected line is entirely different, we can determine that the work is a forgery, and the error rate is also 100%. Some parts of the genuine work and the forgery could match. There are similar images in the results of the genuine work and forgery. However, the images could be distinguished from those of the forgery. As shown in the insets in Fig 6A–6C, the 6G-6F error rates were as large as 90%. Thus, it is possible to authenticate the genuine paintings with high reliability.

### The optical measurements for creating the database

**Fig 7:**
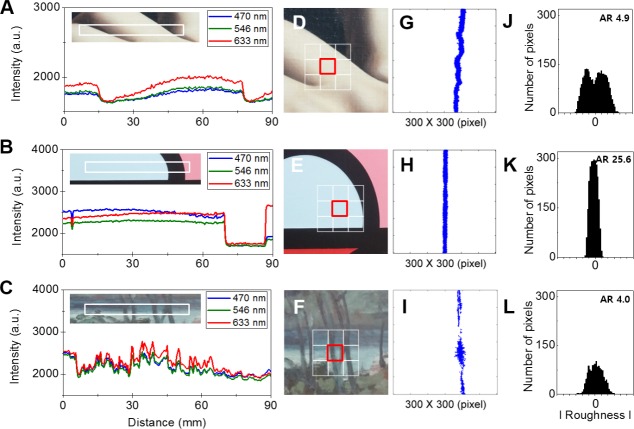
Measurement data for creating the database. (A), (B) and (C) are the measured RGB color spectra of the paintings in the white boxes. (D), (E) and (F) present parts of the artworks. The red boxes indicate the scanned regions; (G), (H), and (I) are the obtained topographic images using the threshold process (300 × 300 pixels); (J), (K) and (L) are the numbers of pixels along roughness and presented with the ARs.

To document precious genuine paintings, we developed a method for creating a database of genuine paintings. Fig 7 shows the data in specific area of the three genuine paintings. The spectra in Fig 7A–7C were selected from the white rectangular boxes. The spectra in Fig 7A are the results from the region in the genuine *Self-portrait*. There are two major characteristics: 1) the measured intensity at 633 nm was generally higher than those of the others (470 nm and 546 nm) and 2) all the spectra in the dark regions were diminished. The measured data from the genuine *Morpheme* are shown in Fig 7B, and the results show that the artwork was painted almost evenly. All the spectral intensities measured in the black region were also decreased, and the 633 nm spectrum was notably increased in the pink region. The particular picture of the genuine *Unknown* shows the river and trees, and the frequent color intensity changes are represented as frequent changes in the spectra. We also measured the reflections in the portions of the three genuine paintings shown in Fig 7D–7F. Fig 7G–7I are the topographic images measured in the red boxes of Fig 7D–7F. The line in Fig 7G is slightly tilted and rough, which means that the surface of that part of the painting is uneven. In contrast, as shown in Fig 7H, the detected line is straight and almost flat, which indicates that the surface is almost flat. The height of the painting varies little owing to the pigments and brushstrokes. There is a little scattering in the line in Fig 7I. Fig 7J–7L show the calculated graphs, which were derived from Fig 7G–7I, respectively. The graph in Fig 7J shows bifurcation and extension. Fig 7K is relatively straight and taller than the other graphs because the reflection image is comparatively straight and sharp. In the graph shown in Fig 7L, the number of pixels is lower than those of the others because the quantity of detected light was generally less and more light was scattered. We also indicated the ARs of the lines in the processed images in Fig 7J–7L. The AR values in Fig 7J–7L were 4.9, 25.6, and 4.0. The AR in Fig 7K is higher because of the sharp reflected line. These values can serve as the numerical information for the topographies of the paintings.

**Fig 8:**
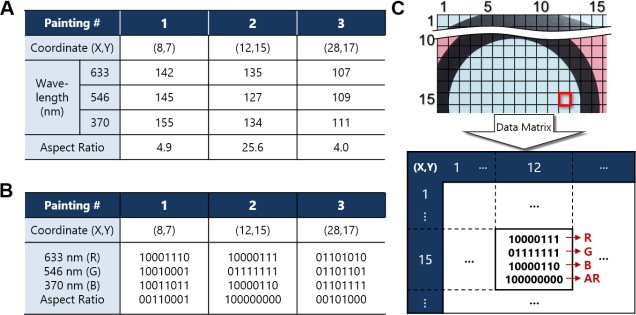
Processing for creating the database. An example of creating the database of the genuine works. (A) The decimal database of the red boxes in Fig 3D, 3E and 3F. (B) The converted binary numerical values from the decimal numbers. (C) The resulting matrix database for painting 2.

Fig 8 shows the process for creating the database. First, the target painting was divided into several sections of 1 × 1 cm, and the sites were defined in a matrix; painting #1 was divided into 600 sections, painting #2 was divided into 1900 sections, and painting #3 was divided into 1200 sections. Each section contains location information expressed as coordinates. The data in each section are the characteristic information of the color and topography in sequence. The color and topography information was represented by the intensities and ARs in Fig 8A. The intensities of color information at each wavelength were converted from 0 to 255 to decimal numbers, which were measured in the center of each section. The decimal data were also converted into binary numerals, as shown in Fig 8B. The converted binary values were encoded into matrix form to build the database. All the unique characteristics of the paintings can be concisely stored as a light text file. We can identify the database that we want to test simply by searching the information for the artwork and then by finding the scanning location of each painting. The stored database, as illustrated in Fig 8C, can then be used for authenticity tests.

## Discussion

We confirmed the feasibility of using a simple and accurate optical measurement system to distinguish forgeries from genuine works and created an authenticity database. The RGB spectra and topographic information about the paintings were obtained via an optical measurement, and the measured data at the micron scale were then encoded and saved in the database. The measurement data show spectral differences between the genuine paintings and the forgeries even though the originals were precisely imitated by experts. The data provided clear information that could be used to distinguish the genuine works from the forgeries. The advantage of combining two techniques is that the characteristics of the all surface of the paintings about both color and appearance can be observed with this simple system. Additionally, we think that the combined system become complementary approach to analyze the painting. We also conducted blind tests on the works using the database to prove the accuracy of our system for determining authenticity. Consequently, the testers were able to distinguish the forgeries and originals with 100% accuracy. It is possible to distinguish the genuine painting and the forgery even if one of the results is compared as we showed results. If needed, the comparison of whole data is also possible to distinguish paintings accurately. However, it spends more time when the size of paintings gets larger. Generally, we select and compare the data about 5 × 5 cm region for the effective authenticity.

It is also available to digitize the information of the painting as it is represented. The database of the paintings will prevent controversies over authenticity. When similar paintings appear, it will be possible to identify the genuine work by comparing with the data. In addition, long after a genuine work has been naturally weathered or physically or chemically damaged, the database can be used as a guide for repairing the damaged regions on the basis of the genuine data. In the future, we hopefully will analyze the most famous works and create databases for them. However, some factors such temperature, humidity and dust can affect the paintings. For example, levels of humidity can affect the contraction and expansion of the paintings, so the surface of paintings could be changed. The artworks should be stored will in appropriate conditions. If not, the removal of dusts on the surface should be primary processed before evaluation for the accurate measurements. If the paintings get deformed from humidity or changes of environmental temperature, a regular-term of measurement is good solution because the deformation does not be processed in a single day. If the paintings are outdated without severe damage, the color of paintings would be faded slowly. The changes of RGB spectral intensities may be gradually reduced in general or partial area. The spectral changes can be also revised enough through the annual spectral measurements. In addition, the positions where the peaks of RGB spectra change are considered to be comparable.

## Supporting information

S1 Fig The RGB spectra for comparison of the scanning speed. The RGB spectra for comparison of the scanning speed. We obtained the scanned results in the area with the white line. We can undoubtedly distinguish the original work from a forgery at a regular speed of 0.09 cm/s (A and C). When the painting was scanned at a speed of 0.9 cm/s for quick authentication, which is 10-fold faster than 0.09 cm/s, the RGB spectra in B and D were obtained. Those data present monotonic lines, but they still have the characteristics of the paintings that are shown in graphs A and C. We were able to thoroughly compare the data for the original and forgery at both scan speeds.
(TIF)

S2 Fig The optical coherence tomography (OCT) setup for measuring the paintings. We designed optical coherence tomography (OCT) for measuring tomography of the paintings.
(TIF)

S3 Fig The images from the genuine works and forgeries in the white boxes. This figure shows images of the scanned data from the genuine works and forgeries in the white boxes. The scattered light was removed by setting a threshold, and we can observe that all of the detected images were different. A, D, F data in Fig 4 is based on these images. The topography in each area presents various characteristics.
(TIF)

S4 Fig The comparison data in the same area. The comparison data that were scanned three times in the same area. The three results for each wavelength were almost equivalent.
(TIF)

S5 Fig The measured image from random areas on the forgery and the original *Self-portrait*. The measured reflected light detected from randomly picked areas on the forgery and the original *Self-portrait*. A-E designate the randomly picked areas of the paintings. Two executions measured at the same position on the original work are indicated as E1 and E2. The E1 and E2 data appear to be similar; however, the forgery data were totally different.
(TIF)
