# Supplementary figures and images for: Optical measurements of paintings and the creation of an artwork database for authenticity
Source: PLoS One. 2017 Feb 2;12(2):e0171354. doi: 10.1371/journal.pone.0171354 (PMC5289567; doi:10.1371/journal.pone.0171354)

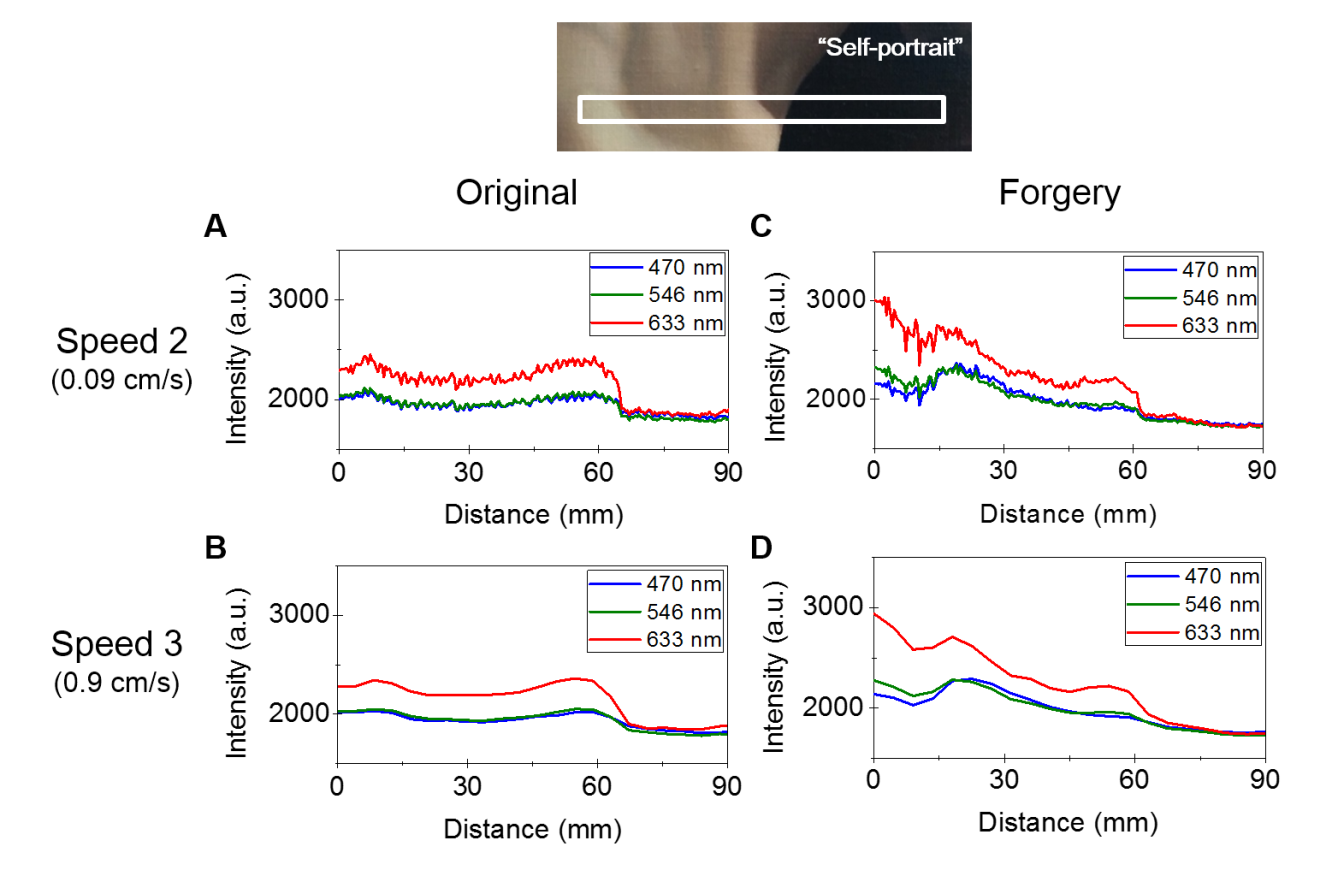

Supplement: S1 Fig — The RGB spectra for comparison of the scanning speed. We obtained the scanned results in the area with the white line. We can undoubtedly distinguish the original work from a forgery at a regular speed of 0.09 cm/s (A and C). When the painting was scanned at a speed of 0.9 cm/s for quick authentication, which is 10-fold faster than 0.09 cm/s, the RGB spectra in B and D were obtained. Those data present monotonic lines, but they still have the characteristics of the paintings that are shown in graphs A and C. We were able to thoroughly compare the data for the original and forgery at both scan speeds. (TIF) [file pone.0171354.s001.tif]

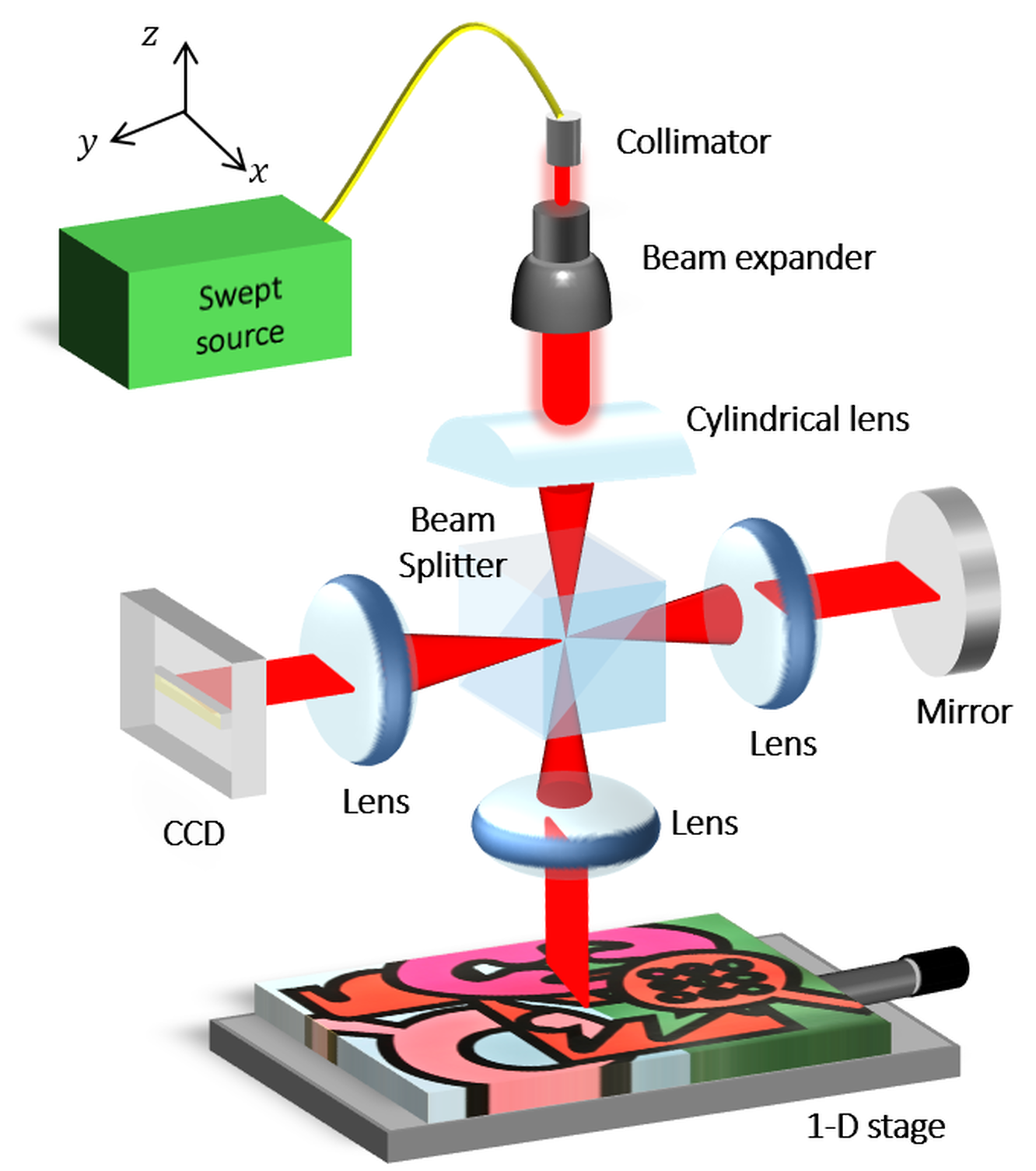

Supplement: S2 Fig — We designed optical coherence tomography (OCT) for measuring tomography of the paintings. (TIF) [file pone.0171354.s002.tif]

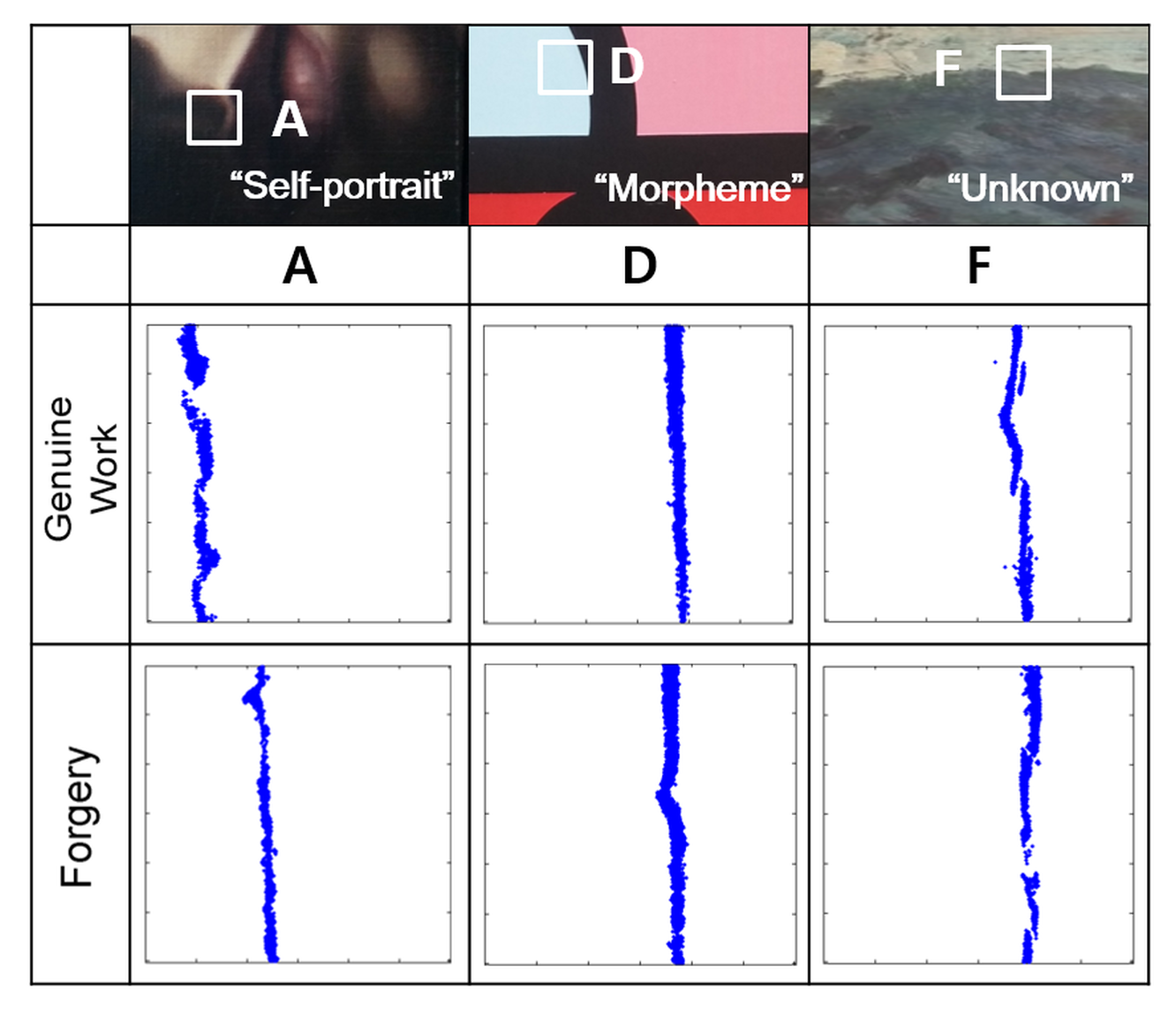

Supplement: S3 Fig — This figure shows images of the scanned data from the genuine works and forgeries in the white boxes. The scattered light was removed by setting a threshold, and we can observe that all of the detected images were different. A, D, F data in Fig 4 is based on these images. The topography in each area presents various characteristics. (TIF) [file pone.0171354.s003.tif]

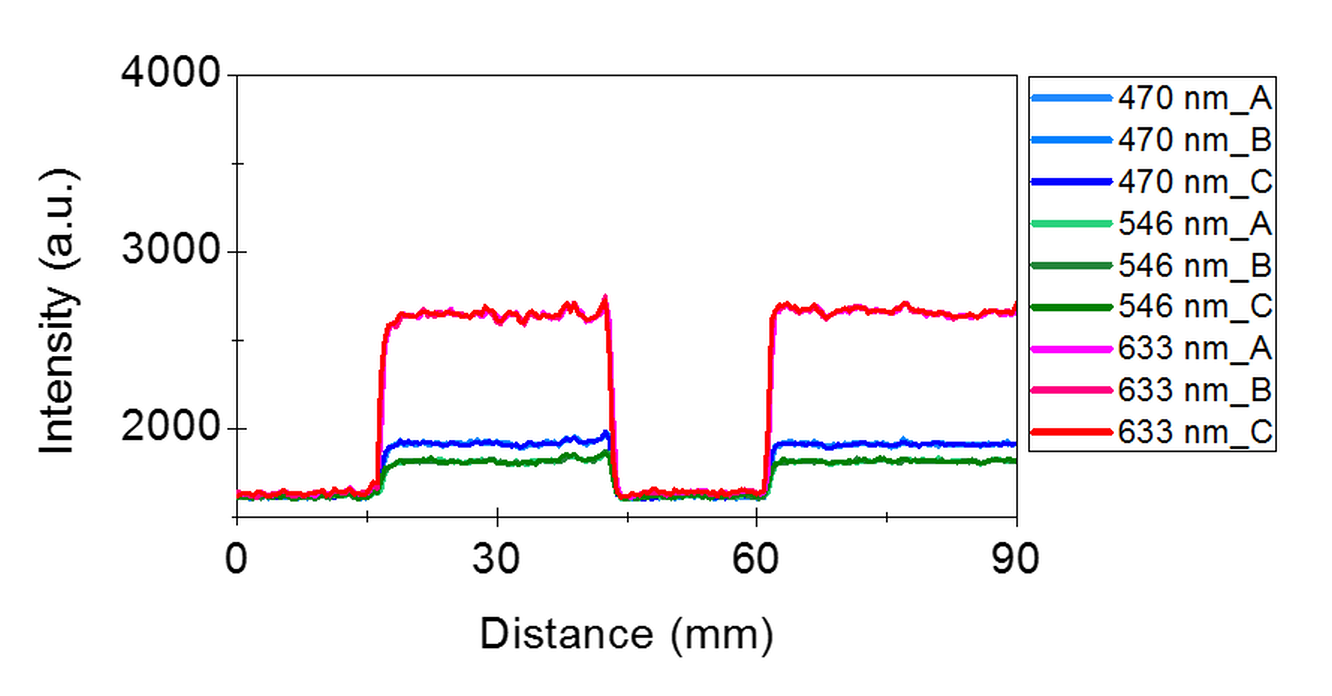

Supplement: S4 Fig — The comparison data that were scanned three times in the same area. The three results for each wavelength were almost equivalent. (TIF) [file pone.0171354.s004.tif]

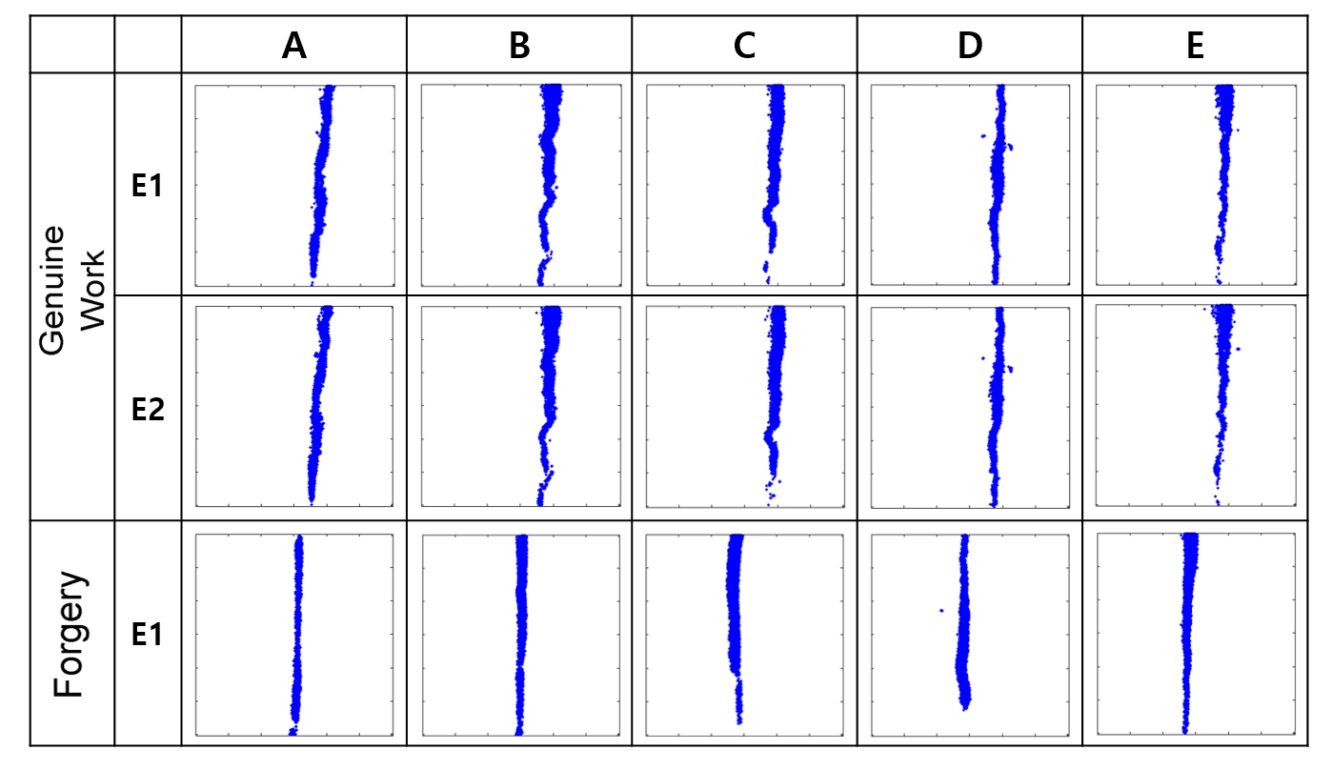

Supplement: S5 Fig — The measured reflected light detected from randomly picked areas on the forgery and the original Self-portrait. A-E designate the randomly picked areas of the paintings. Two executions measured at the same position on the original work are indicated as E1 and E2. The E1 and E2 data appear to be similar; however, the forgery data were totally different. (TIF) [file pone.0171354.s005.tif]
